# Telomere length differences between colorectal polyp subtypes: a colonoscopy-based case-control study

**DOI:** 10.1186/s12885-018-4426-2

**Published:** 2018-05-02

**Authors:** Sheetal Hardikar, Andrea N. Burnett-Hartman, Amanda I. Phipps, Melissa P. Upton, Lee-Ching Zhu, Polly A. Newcomb

**Affiliations:** 1 Huntsman Cancer Institute, University of Utah, 2000 Circle of Hope Dr. Room 4711, Salt Lake City, UT 84112 USA; 2 Public Health Sciences Division, Fred Hutchinson Cancer Research Center, Seattle, WA USA; 3 Kaiser Permanente, Colorado Institute for Health Research, Denver, CO USA; 4 Department of Epidemiology, School of Public Health, University of Washington, Seattle, WA USA; 5 Department of Pathology, School of Medicine, University of Washington, Seattle, WA USA; 6 Kaiser Permanente Washington Health Research Institute, Seattle, WA USA

**Keywords:** Adenomas, Serrated polyps, Sessile serrated polyps, Advanced adenomas, Telomere length

## Abstract

**Background:**

Short telomeres have been associated with increased risk of many cancers, particularly cancers of the gastrointestinal tract including esophagus and stomach. However, the association between telomere length (TL) and colorectal cancer and its precursors, colorectal polyps, is not clear.

**Methods:**

We investigated the relationship between TL and risk of colorectal polyp subtypes in a colonoscopy-based study in western Washington. Participants were 35–79 year-old enrollees at an integrated health care system, who underwent a colonoscopy between 1998 and 2007 (*n* = 190), completed a self-administered questionnaire, provided blood samples, and were distinguished as having adenomas, serrated polyps, or as polyp-free controls through a standardized pathology review. Telomere length (T) relative to a single copy gene (S) was measured in circulating leukocytes from stored buffy coat samples using quantitative polymerase chain reaction. Multivariable polytomous logistic regression was used to compare case groups with polyp-free controls and other case groups; adjusted odds ratios (OR) and 95% confidence intervals (CI) were estimated.

**Results:**

TL in the shortest tertile (T/S ratio < 0.58) was associated with increased risk of adenomas and serrated polyps [OR (95%CI) were 1.77(0.81–3.88) and 2.98(1.15–7.77), respectively). When evaluated by lesion severity within each pathway, short TL was more strongly associated with advanced adenomas and sessile serrated polyps [OR (95% CI) = 1.90(0.76–4.73) and 3.82(0.86–16.86), respectively], although the associations were not statistically significant.

**Conclusions:**

Our results suggest that short TL may be associated with an increased risk of colorectal polyps in both the adenoma-carcinoma and serrated pathways. The risk was particularly notable for sessile serrated polyps, although the association was not statistically significant and sample size was limited.

## Background

Colorectal cancer (CRC) is a multi-pathway disease; unique CRC pathways are associated with distinct polyp precursor lesions. Advanced adenomas are known precursor lesions to CRC within the adenoma-carcinoma pathway, while sessile serrated adenomas/polyps (SSA/Ps), are precursors on the serrated pathway [1]. Currently, colorectal polyps are not diagnosed until colonoscopy, an expensive and invasive procedure. Moreover, advanced adenomas are diagnosed in about 6% of those undergoing colonoscopy; rate of SSA/P diagnosis is lower [2]. If reliable biomarkers that enable risk stratification to identify those at highest risk for progression to CRC can be identified, patients could be triaged to tailored screening and surveillance regimes that coincide with their level of risk.

One such potential biomarker of interest is telomere length (TL). Telomeres, the DNA repeat sequences at ends of chromosomes, play an important role in maintaining genomic integrity [3]. Short TL has been evaluated as a biomarker for ageing, and age-related conditions, including cancers; particularly of the gastrointestinal tract [4]. The relationship between shortened telomeres and risk of sporadic colorectal cancers, however, is not yet clear. Here, we evaluate the association between TL and the risk for colorectal polyp subtypes.

## Methods

### Study population

The study population for the current project was derived from a parent study within Kaiser Permanente Washington, a large integrated healthcare provider in western Washington state. Details of the parent study have been previously published [5]. We selected 35–79 year old enrollees who had undergone an index colonoscopy for any indication from 1998 to 2003 (phase 1) and 2004–2007 (phase 2), and had available stored genomic DNA (*N* = 190). Participants with a previous colonoscopy within the past year, a history of CRC or other colorectal diseases, familial colorectal cancer syndromes, and past partial resection of the colon were excluded. Written informed consent was obtained from all study participants and institutional review boards at both Fred Hutchinson Cancer Research Center and Kaiser Permanente Washington approved the study.

### Data collection and case/control ascertainment

Participants in both phases completed a structured questionnaire to collect information on risk factors, and medical, family, and colorectal cancer screening history. Biopsies taken at the time of index colonoscopy received a standardized pathology review, as described elsewhere [5]. Participants were diagnosed as having adenomas (tubular, villous or tubulovillous adenomas), serrated polyps (hyperplastic polyps, traditional serrated adenomas, or SSA/Ps), or both. Polyp-free controls were identified during index colonoscopy, and reflected the age distribution and diagnosis year of the polyp cases. Adenomas ≥10 mm in diameter or with ≥20% villous components or exhibiting high-grade dysplasia were classified as advanced adenomas (non-advanced otherwise). SSA/Ps were considered as advanced lesions, while hyperplastic polyps were considered to be non-advanced lesions in the serrated polyps subgroup.

### Telomere length measurement

Genomic DNA was extracted from lymphocytes (phase 1) or buccal DNA (phase 2) using the Qiagen QIAamp DNA extraction kit (Qiagen Inc., Valencia, CA) and stored at − 80 °C. Telomere length (T) relative to a single copy gene 36B4 (S) was measured using a singleplex quantitative polymerase chain reaction (qPCR), as described by Cawthon [6]. All assays were run in triplicate and a standard laboratory control was run on each plate. Each plate run was then assessed for efficiency and precision (R2) using the standard curve generated using the laboratory control. R2 values for the telomere and control gene standard curves were greater than 0.98, demonstrating great precision. Efficiency for the telomere and control gene PCRs were between 99% and 110%. The mean normalized T/S ratio was used for the statistical analysis.

### Statistical analyses

**Fig. 1:**
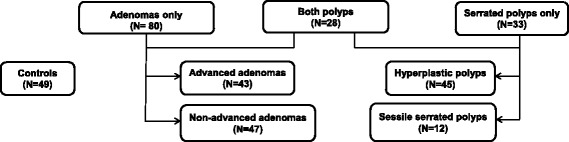
Study participants’ outcomes based on index colonoscopy

Multivariable polytomous logistic regression was used to compare case groups (adenomas only, serrated polyps only, or both) with each other and with polyp-free controls. Odds ratios (OR) and 95% confidence intervals (CIs) for short TL (shortest telomere tertile; T/S ratio < 0.58) were computed adjusting for age, sex, race, smoking status, body mass index, and use of anti-inflammatory drugs These factors were selected a priori based on evidence from prior studies reporting associations between them and TL or colorectal polyps. Wald *p*-values were calculated to compare heterogeneity between case groups. Analyses evaluating associations between TL and lesion severity included persons with synchronous adenomas and serrated polyps within both the adenoma as well as serrated pathway groups (Fig. 1), because we were interested in evaluating the differences between advanced and non-advanced lesions within a particular pathway, irrespective of the presence of polyp lesions from another pathway. All analyses were performed using Stata (v14.0; Stata Corp).

## Results

Of the 190 study participants with available DNA specimens, 80 were classified as having adenomas, 33 as serrated polyps, 28 as having both adenomas and serrated polyps, and were compared to 49 polyp-free controls. Demographic comparisons between polyp subtype cases and controls are shown in Table 1. Participants with adenomas only were slightly older and more likely than controls to be male and former smokers. In contrast, study participants with serrated polyps only were more likely to be female, current/former smokers, and obese. Cases were similar to controls with respect to other lifestyle exposures such as their educational status, and anti-inflammatory drug use.

**Table 1:** Participant characteristics by polyp status, Kaiser Permanente Washington, Seattle, Washington, 1998-2007

Patient characteristics		Controls	Adenomas	Serrated polyps	Both Adenomas + Serrated polyps
		(*n* = 49)	(*n* = 80)	(*n* = 33)	(*n* = 28)
Age in years; Mean (SD)		59.7 (10.8)	62.3 (10.4)	61.7 (9.2)	62.3 (10.9)
Gender; n(%)	Male	25 (51)	44 (55)	13 (39.4)	13 (46.4)
	Female	24 (49)	36 (45)	20 (60.6)	15 (53.6)
White race; n(%)		44 (89.8)	72 (90)	29 (87.8)	25 (89.3)
Smoking; n(%)	Never	20 (40.8)	27 (33.8)	9 (27.3)	9 (32.1)
	Former	22 (44.9)	50 (62.5)	20 (60.6)	14 (50)
	Current	7 (14.3)	3 (3.8)	4 (12.1)	5 (17.9)
Alcohol use; n(%)		31 (63.3)	44 (55)	22 (66.7)	18 (64.3)
BMI; n(%)	< 25	17 (34.7)	25 (31.3)	10 (30.3)	8 (28.6)
	25–29.99	19 (38.8)	37 (46.3)	10 (30.3)	9 (32.1)
	30+	13 (26.5)	17 (21.4)	13 (39.4)	11 (39.3)
Education; n(%)	<High school	8 (16.3)	11 (13.8)	4 (12.1)	3 (10.7)
	Some college	11 (22.5)	21 (26.3)	9 (27.3)	12 (42.9)
	College +	30 (61.2)	48 (60)	20 (60.6)	13 (46.4)
Anti-inflammatory drug use; n(%)		26 (53.1)	41 (51.3)	17 (51.5)	10 (35.7)
Physical activity ≥60 MET h/wk.; n(%)		41 (83.7)	62 (77.5)	26 (78.8)	23 (82.1)
Relative Telomere length; Median (IQR)		0.60 (0.56–0.72)	0.60 (0.50–0.71)	0.57 (0.45–0.67)	0.59 (0.51–0.67)
Short telomeres; n(%)	T/S < 0.58 or shortest tertile^a^	17 (34.7)	36 (45.6)	19 (57.8)	14 (50)

*BMI* Body Mass Index, *MET* Metabolic Equivalent of Task

^a^Telomere tertiles were based on the telomere length distribution among controls only

Table 2 presents the ORs and 95% CI for each colorectal polyp subtype outcomes according to shortest telomere tertile. Having TL in the shortest tertile (T/S ratio < 0.58) was associated with an increased risk of serrated polyps [adjusted OR(95%CI) =2.98 (1.15,7.77)] even after adjustment for confounding effects of age, sex, race, smoking status, body mass index, and use of anti-inflammatory drugs. Short TLs were also modestly and non-significantly associated with increased adenoma risk [OR(95% CI) =1.77 (0.81,3.88)] in adjusted models. When evaluated by lesion severity within each pathway, short telomeres appeared to be more strongly associated with advanced adenomas [OR(95% CI) = 1.90(0.76,4.73)] and sessile serrated polyps [OR(95% CI) = 3.82(0.86,16.86)], although these associations did not reach statistical significance. Lastly, we did not find any evidence for heterogeneity in the associations with short TL for adenomas vs. serrated polyps (p for heterogeneity =0.42) or for lesion severity comparisons within the adenoma and serrated pathways.

**Table 2:** Associations between shortest telomere tertile and colorectal polyp subtypes

	Telomere tertile^a^ 2 & 3	Telomere tertile^a^ 1		
	T/S ratio > =0.58	T/S ratio < 0.58		
	*N* (%)	*N* (%)	Unadjusted OR	Adjusted^b^ OR
*Adenoma-Serrated Outcome*				
Controls	32 (65.3)	17 (34.7)	REF	REF
Adenomas	43 (54.3)	36 (45.6)	1.58 (0.75,3.29)	1.77 (0.81,3.88)
Serrated	14 (42.4)	19 (57.6)	2.55 (1.03,6.33)	2.98 (1.15,7.77)
Both	14 (50.0)	14 (50.0)	1.88 (0.73,4.85)	1.94 (0.72,5.27)
*P for heterogeneity between Adenomas & Serrated = 0.42*				
*Adenoma Outcome*				
Controls	32 (65.3)	17 (34.7)	REF	REF
Non-advanced adenoma	25 (54.4)	21 (45.7)	1.58 (0.69,3.61)	1.63 (0.69,3.86)
Advanced adenoma	22 (51.2)	21 (48.8)	1.80 (0.78,4.16)	1.90 (0.76,4.73)
*P for heterogeneity between Advanced & Non-advanced adenomas = 0.71*				
*Serrated Outcome*				
Controls	32 (65.3)	17 (34.7)	REF	REF
Hyperplastic polyps	22 (48.9)	23 (51.1)	1.97 (0.86,4.51)	2.24 (0.88,5.70)
Sessile serrated polyps	4 (33.3)	8 (66.7)	3.76 (0.99,14.3)	3.82 (0.86,16.86)
*P for heterogeneity between Hyperplastic & Sessile Serrated Polyps = 0.61*				

a Telomere tertiles were based on the telomere length distribution among controls only

^b^Models adjusted for age, gender, race, BMI, smoking, and use of anti-inflammatory drugs

## Discussion

There are limited prior studies evaluating the association between shortened telomeres and colorectal neoplasia. Two case-control studies nested within large cohorts observed a lack of association between TL and CRC [7–9]. Conversely, Pooley et al. have reported significantly shorter telomeres in circulating leukocytes among CRC cases than controls [10]. More recently, a large population-wide Danish study of over 47,000 individuals reported no association, while a Chinese study reported increased CRC risk with shorter TL [9, 11]. Summarizing these findings, a very recent meta-analysis noted that the available evidence is insufficient to understand the exact role of telomere length in the development of colorectal cancer, and highlighted the need for future studies [12]. Fewer studies have evaluated the relationship between colorectal cancer precursor lesions and TL. A recent report suggested an increased risk for advanced adenomas in persons with short leukocyte TL [13]; however, this study was relatively small (35 advanced adenomas cases) and did not evaluate the relationship with serrated polyps. Roger et al. reported in an experimental setting that extensive tissue telomere erosion may lead to chromosomal instability and initiation of CRC in polyps from familial adenomatous polyposis patients [14]. The authors hypothesize that this may be a potential mechanism resulting in chromosomal instability and malignant transformation. Another cross-sectional study found that TL in large adenoma lesions (> 2 cm) was significantly shorter than normal surrounding tissue from the same individuals [15]. Taken together, these studies suggest that telomere shortening may play a role in the development of both colorectal polyps and/or CRC. However, further studies are required to better understand the role of TL (and the point at which they may act) in the pathological progression in colorectal neoplasia.

Our analysis is an important addition to the literature, because it is the first study to report a statistically significant association of TL with serrated polyps, suggesting that telomeres may play an important role along the entire serrated pathway. There was also a suggestion of increased risk, particularly, for SSA/Ps. SSA/Ps are newly-recognized precursors to CRC [16]; thus, this finding may provide valuable insights into the underlying mechanisms for cancer progression within the serrated pathway. Further work is warranted to determine whether TL can serve as a reliable biomarker for such a neoplastic progression.

Our study is larger than most other studies of colorectal polyps and TL, but it is nonetheless limited by sample size, particularly as colorectal polyp cases were further divided into smaller subgroups based on lesion severity. However, this enabled us to look at differences within sub-classes of colorectal polyps. Also, misclassification of disease status in some cases and controls is possible, particularly with respect to the harder to visualize SSA/Ps. However, such misclassification would be non-differential and would bias estimates towards the null. Hence, our reported positive association with serrated polyps is conservative. Finally, although we have controlled for major potential confounders, including age, sex, smoking, body mass index, and inflammation, we cannot exclude the possibility of residual confounding by measured and unmeasured risk factors. Strengths of our study include the high-quality and detailed characterization of colorectal polyp cases and controls using colonoscopy and standardized pathology review, which enabled us to evaluate the relationship between short telomeres and various polyp subtypes. Additionally, to our knowledge, this is the first study to report associations between short telomeres and advanced polyps within the serrated pathway.

## Conclusions

In conclusion, short telomeres were associated with an increased risk of colorectal polyps in both the adenoma-carcinoma and serrated pathways, although the increased risk was statistically significant only for serrated polyps. Among individuals with serrated polyps, the risk associated with short telomeres appeared stronger, but not statistically significant, for those with SSA/Ps. Future prospective studies are needed to define the temporal sequence between TL and these high risk lesions, and to elucidate the role of telomeres as a biomarker for CRC risk stratification.

## Abbreviations

CI: Confidence interval
CRC: Colorectal cancer
OR: Odds ratio
SSA/P: Sessile serrated adenoma/polyp
TL: Telomere length
